# Latent profile analysis of burnout, depression, and anxiety symptoms among Chinese medical staff with frontline anti-epidemic experience in the post-COVID-19 pandemic era

**DOI:** 10.3389/fpubh.2024.1458167

**Published:** 2025-01-06

**Authors:** Huachun Xu, Lanjun Qiu, Yu Chen, Mengjun Zhang, Junyi Li, Guochun Xiang

**Affiliations:** ^1^College of Psychology, Sichuan Normal University, Chengdu, China; ^2^School of Public Health, Southern Medical University, Guangzhou, China

**Keywords:** latent profile analysis, burnout, depression, anxiety, medical staff

## Abstract

**Background:**

Frontline medical staff’s psychological symptoms deserve persistent attention after 3 years of high-pressure and high-intensity work during the pandemic. In addition, the meaning of burnout and its relationship with depression and anxiety have long been debated. This study aimed to identify profiles of these symptoms among Chinese medical staff with frontline anti-epidemic experience, along with their distinguishing characteristics.

**Methods:**

Psychological symptoms of burnout (exhaustion, cynicism, and inefficacy), depression, and anxiety from 989 doctors and 1,105 nurses were explored with latent profile analysis. The R3step method was conducted to analyze the predictive factors of those medical staff’s symptoms.

**Results:**

Three symptom profiles were identified for medical staff, with high-level (62.1%), moderate-level (28.9%), and low-level symptoms (9.0%). In the low-level and moderate-level profiles, symptom variables considered had a consistent trend. However, within the high-level profile, the inefficacy of burnout remained at a lower level, and anxiety performed as the most prominent symptom. Variables of gender, age, salary satisfaction, work hours, and work intensity predicted medical staff profiles (*p* < 0.05).

**Conclusion:**

In the post-COVID-19 era, former frontline Chinese medical staff’s psychological symptoms were divided into three latent profiles. Symptoms of burnout, depression, and anxiety did not move in lock-step, indicating that they are different and robust constructs. Targeted intervention strategies should be developed for different subgroups.

## Introduction

During the COVID-19 pandemic, frontline medical staff was faced with an extremely uncertain work environment and high-intensity work pressure, which led to a high prevalence of psychological symptoms among them (1–3). Further, the experience of fighting the epidemic on the front line could have been traumatic, and the related psychological symptoms may persist for long periods. Previous evidence has shown that the experience of treating SARS patients during the SARS outbreak brought long-term psychological and occupational impact, manifested as significantly higher levels of psychological symptoms (such as burnout, posttraumatic stress, depression, and anxiety) compared to those healthcare workers without the experience, even 1 or 2 years later (4, 5). Therefore, despite being in the post-pandemic era after January 8, 2023, when China entered the regular epidemic prevention and control stage, the long-term impact of the 3 years during the pandemic on the psychological symptoms of medical staff with frontline anti-epidemic experience deserves concern.

Numerous studies have suggested that burnout is a global phenomenon among medical staff. Burnout is conceptualized as a stress-related problem for individuals who work in interpersonally oriented occupations such as healthcare (6, 7), and consists of three interrelated components: exhaustion, cynicism, and inefficacy. Besides, depression and anxiety have always been challenging problems before and during the COVID-19 defense in China (8–11).

On the other hand, the meaning of burnout and its relationship with depression and anxiety have long been debated in academia (12). For example, previous literature reviews focusing on the distinction between burnout and depression have yielded mixed conclusions (13, 14). Especially, a growing body of research has supported that burnout and depression are overlapping constructs. Evidence suggests these concepts refer to syndromes with blurred boundaries, which often show a high degree of comorbidity and simultaneous onset (13). Burnout symptoms share a general dimension with depression (15), and they seem to represent the same continuum of distress (16). All of this evidence casts serious questions as to whether burnout is a standalone syndrome independent of depression. However, although burnout and depression appear to share some common features (e.g., loss of vitality), several researchers believe that burnout and depression are two separate constructs (17). At the same time, some researchers held that burnout can only be experienced in reaction to stressors experienced in the workplace, while depression is more pervasive and not specific to the work context (18, 19). Also, some researchers found that burnout could significantly predict depression (20, 21), and some other researchers believed that depression can increase the possibility of burnout (22). Whereas, other studies suggested a reciprocal relationship (17, 23). Similar controversies also exist regarding the relationship between burnout and anxiety (14, 24–26). Ding et al. (2014) found that exhaustion and cynicism were positively related to anxiety symptoms, whereas inefficacy was negatively related to anxiety symptoms (27). Turnipseed also found that there was a significant correlation between burnout and anxiety symptoms, with the connection between anxiety and exhaustion being the strongest (28). However, the exact relationship between burnout and anxiety is unclear. Specifically, are people with higher anxiety more prone to burnout, or does burnout exacerbate anxiety symptoms? Furthermore, is there an overlap between burnout and anxiety? Accordingly, more research is needed to understand how burnout relates to depression and anxiety symptoms among medical staff.

By now, most of the previous studies investigating burnout, depression, and anxiety symptoms together have adopted the “variable-centered” approach, assuming that all individuals from a sample are drawn from a single population for which a single set of “averaged” parameters can be estimated (29, 30). To the best of our knowledge, the person-centered approach has not been applied to study the relationship between the three components of burnout and symptoms of depression and anxiety. LPA is an emerging person-centered analytical technique that classifies individuals based on model-fitting estimation. Unlike traditional methods, LPA does not require strong causal relationships between variables or large sample sizes collected from multiple time points. This statistical approach explains the relationships between observed continuous indicators through latent categorical variables. Objective statistical indices are employed to assess the accuracy and validity of the classification, thereby maximizing the identification of qualitative differences among individuals (31–33). The focus of this approach is on identifying latent sub-populations of individuals based on multiple observed characteristics (i.e., indicators). This provides a higher level of specificity compared to variable-centered approaches, offering a more intuitive and realistic description of the individuals and enhancing our understanding of the subjects (34).

With the construct of burnout, Leiter and Maslach have used latent profile analysis and identified multiple person-centered profiles across the burnout–engagement continuum with two large datasets, which demonstrated that dimensions of burnout did not rise and fall together all the time. Accordingly, we may reasonably assume that the relationship between burnout dimensions and symptoms of depression and anxiety do not always move in lock-step. LPA is just the method that can detect the presence of patterns of several variables within individuals that tend to recur between individuals, rather than the effects of specific variables on individuals (35–37), and therefore provides new ways to solve the problem of symptom classification.

Previous studies have shown that demographic variables and work-related variables have significant effects on medical staff’s psychological symptoms. For example, female medical staff tended to show more burnout and anxiety symptoms (38) and were more likely to suffer from depression than men (39); compared to married medical staff, unmarried nurses were more prone to burnout (40); workers in secondary hospitals were more likely to experience depression and anxiety than were those working in tertiary hospitals (41); medical staff with fewer work years and little practical experience were more likely to burnout (40); and medical staff who had low salary satisfaction and more job dissatisfaction had greater burnout (42). Accordingly, it is also meaningful to account for demographic and work-related factors when examining psychological symptoms via LPA. Especially, the results will help address the profile members with a high risk of psychological problems (43).

In summary, frontline medical staff may continuously experience burnout, depression, and anxiety after a sustained and high-intensity public health emergency (4, 5, 44). The present study extends previous research by assessing burnout, depression, and anxiety jointly with latent profile analysis. This analysis will help in accurately discerning the heterogeneity of these symptoms and understanding the relations between them in medical staff. Accordingly, this study aimed to investigate burnout (consists of exhaustion, cynicism, and inefficacy), depression, and anxiety symptoms of Chinese medical staff with frontline anti-epidemic experience through LPA. Also, we explored whether relevant factors (e.g., demographic variables and work-related variables) are significant influencing factors of distinct symptom profiles.

## Methods

### Participants and procedures

For this study, data were collected using an online survey platform, Wenjuanxing (Questionnaire Star), which is widely used for research purposes in China. The survey was completely anonymous and voluntary, ensuring that participants’ responses were confidential and that no personal identifying information was collected. The data collection process followed the ethical guidelines set by our institutional review board, with informed consent obtained from all participants before participation.

The survey was distributed across four provinces of China (Guangdong, Guizhou, Chongqing, and Sichuan), following a stratified random sampling approach to ensure institutional diversity. The survey was open to healthcare professionals working in various departments between February and April 2023, with the following specific inclusion criteria: (1) being a doctor or a nurse; (2) being over 18 years of age; and (3) having participated in the anti-COVID-19 effort as frontline staff. We ensured that each participant could only submit one completed survey online to maintain data integrity.

Finally, a total of 2094 healthcare workers, consisting of 989 doctors and 1,105 nurses participated in the survey. As shown in Table 1, most of the participants were under 35 years of age (*n* = 1,457, 69.6%), worked in public tertiary hospitals (*n* = 784, 37.4%) and community healthcare centers (*n* = 681, 32.5%), were female (*n* = 1,577, 75.3%), were married (*n* = 1,564, 74.7%), had children (*n* = 1,486, 71.0%), had bianzhi (*n* = 1,120, 53.5%); Table 1 also shows the variables of education, job title, work years, income, salary satisfaction, work hours and work intensity.

**Table 1:** Comparisons of medical staff’s characteristics among profiles.

Characteristic	Categories	Total (*n* = 2094)	Profile 1	Profile 2	Profile 3	*χ^2^/F*	*p*
			(*n* = 1,301)	(*n* = 605)	(*n* = 188)		
Position						4.122	0.127
	Doctor	989 (47.2%)	623 (47.9%)	268 (44.3%)	98 (52.1%)		
	Nurse	1,105 (52.8%)	678 (52.1%)	337 (55.7%)	90 (47.9%)		
Work unit						42.609	<0.001
	Public tertiary	784 (37.4%)	483 (37.1%)	226 (37.4%)	75(39.9%)		
	Public secondary	543 (26.0%)	389 (30.0%)	129 (21.3%)	25(13.3%)		
	Community healthcare center	681 (32.5%)	374 (28.7%)	226 (37.4%)	81(43.1%)		
	Private healthcare institution	29 (1.4%)	20 (1.5%)	6 (1%)	3(1.6%)		
	Other	57 (2.7%)	35 (2.7%)	18 (2.9%)	4(2.1%)		
Gender						15.347	<0.001
	Female	1,577 (75.3%)	971 (74.6%)	482 (79.7%)	124(66.0%)		
	Male	517 (24.7%)	330 (25.4%)	123 (20.3%)	68(34.0%)		
Marital status						12.816	0.002
	Single	530(25.3%)	295 (22.7%)	177 (29.3%)	58 (30.9%)		
	Married	1,564(74.7%)	1,006 (77.3%)	428 (70.7%)	130 (69.1%)		
Parenting status						18.816	0.004
	Without children	608(29.0%)	338(26.0%)	206(34.0%)	64(34.0%)		
	With children	1,486(71.0%)	963(74.0%)	399(66.0%)	124(66.0%)		
Bianzhi						3.248	0.517
	Have bianzhi	1,120(53.5%)	693(53.3%)	316(52.2%)	111(59.0%)		
	No bianzhi	974(46.5%)	608(46.7%)	289(47.8%)	77(41.0%)		

Variable	Mean (Standard deviation)				*χ^2^/F*	*p*
Age	4.11 (1.608)	4.21 (1.650)	3.91 (1.509)	4.02 (1.567)	7.563	0.001
Education	3.25 (0.514)	3.25 (0.510)	3.25 (0.532)	3.29 (0.490)	0.61	0.544
Job title	3.64 (0.812)	3.60 (0.826)	3.70 (0.786)	3.70 (0.778)	3.634	0.027
Work years	3.27 (1.205)	3.32 (1.213)	3.18 (1.172)	3.27 (1.244)	2.783	0.062
Income	2.04 (1.134)	2.10 (1.153)	1.95 (1.107)	1.93 (1.065)	4.402	0.012
Salary satisfaction	3.35 (1.121)	3.62 (1.103)	2.91 (0.981)	2.90 (1.085)	112.656	<0.001
Work hours	2.23 (0.672)	2.16 (0.627)	2.28 (0.687)	2.53 (0.810)	22.999	<0.001
Work intensity	3.26 (1.015)	3.06 (1.005)	3.54 (0.897)	3.79 (1.048)	79.486	<0.001

### Measures

#### Burnout

Burnout was assessed via the 22-item Chinese version of the Maslach Burnout Inventory-Human Service Survey (MBI-HSS) (45). The items consist of 3 dimensions: exhaustion (also described as wearing out, loss of energy, depletion, debilitation, and fatigue); cynicism (also described as depersonalization, negative or inappropriate attitudes, detached concern, irritability, loss of idealism, and withdrawal); and inefficacy (also described as reduced productivity or capability, low morale, low personal accomplishment and an inability to cope). A seven-point Likert scale ranging from “never” (1) to “every day” (7) was used to measure the frequency with which respondents experienced feelings related to each item. The Chinese version of MBI-HSS has also been shown to have excellent validity in a sample composed of participants from a range of occupations, including medical staff (46–48). In the present study, the exhaustion scale had an internal consistency (Cronbach’s alpha) of 0.91, the cynicism scale of 0.88, and the inefficacy scale of 0.89.

#### Depression

The Center for Epidemiologic Studies Depression Scale (CES-D) (49) was used to assess depressive symptoms over the past week with 20 items. The responses to the items were given on a four-point Likert scale (never = 1, almost every day = 4). Higher scores on the CES-D indicate more depressive symptoms. Studies of CES-D in different populations, including medical staff, have further confirmed its high adaptability (50, 51). In this study, the Cronbach’s alpha was 0.93.

### Anxiety

The Generalized Anxiety Disorder Scale (GAD-7) (52) is a seven-item scale (GAD1 = Feeling nervous, anxious or on edge; GAD2 = Not being able to stop or control worrying; GAD3 = Worrying too much about different things; GAD4 = Trouble relaxing; GAD5 = Being so restless that it is hard to sit still; GAD6 = Becoming easily annoyed or irritable; GAD7 = Feeling afraid as if something awful might happen), scored on a four-point Likert scale (never = 1, almost every day = 4). The GAD-7 has been shown to exhibit strong reliability and validity across diverse countries and populations (53, 54). In this study, the Cronbach’s alpha was 0.96.

### Demographic and work-related factors

Demographic and work-related variables can influence the psychological symptoms of medical staff. Therefore, we measured work unit (5-point scale; 1 = public tertiary, 5 = other hospitals), gender (1 = female, 2 = male), marital status (1 = single, 2 = married), parenting status (1 = without children, 2 = with children), bianzhi (which is also called the ‘iron rice bowl’ given by the state, meaning positions with lifetime job security; 1 = have bianzhi, 2 = no bianzhi), age (10-point scale; 1 = below 20; 10 = above 61), education (7-point scale; 1 = medical doctor, 7 = primary school or below), job title (5-point scale; 1 = advanced technical job title, 5 = no job title), work years (7-point scale; 1 = less than 1 year, 7 = more than 40), income (9-point scale; 1 = less than RMB 5,000, 9 = more than RMB 200,000), salary satisfaction (5-point scale; 1 = strongly dissatisfied, 5 = strongly satisfied), work hours (4-point scale; 1 = less than 8 h, 4 = more than 12 h), and work intensity (5-point scale; 1 = strongly relaxed, 5 = strongly intensive) using an open-ended response.

### Data analysis

First, Pearson’s *r* correlations for each administered measure among medical staff using SPSS 25.0, and the variables were positively correlated with each other. At the same time, to facilitate clear interpretation continuous scores from psychological symptoms screening tools were standardized by z-score.

Second, the LPA was conducted using Mplus8.3 with a three-step approach (55) to identify profiles of medical staff. In the first step of the LPA, three models were built and tested using the maximum likelihood with robust standard errors (MLR) estimator. In the second step, to identify the appropriate number of latent profiles, we used the Bayesian Information Criterion (BIC), the Akaike Information Criterion (AIC), and the sample-size adjusted BIC (aBIC) (56). Lower values of these indices suggest a better model fit. Additionally, the Lo–Mendell–Rubin adjusted LRT test (LMR-LRT), and bootstrap likelihood ratio test (BLRT) were used to determine the optimal number of profiles. A significant *p*-value for these tests suggested that a K-profile model fit the data better than a model with a (K-1)-profile (57). Meanwhile, when selecting the best model, entropy which checks whether the latent model can represent the different types, is also taken into account. Entropy calculations close to 1.0 indicate a better classification (58).

Finally, a univariate analysis (Chi-square analysis and ANOVA analyses) was conducted to explore the significant associations between sample characteristics (demographic and work-related factors) and psychological symptom profiles. The study then conducted a multinomial logistic regression, using the three-step method implemented by Mplus8.3 through the “Auxiliary” (R3STEP) command to determine the impact of demographic and work-related factors on profile membership. Consistent with previous work, significant factors from the univariate analysis are included in the multinomial logistic regression analysis.

## Results

### Latent profile analysis of medical staff

We assessed the fit of four models and the statistical fit indices are shown in Table 2. The AIC, BIC, and aBIC decreased with an increasing number of latent profiles; both the LMR and BLRT were also significantly less than 0.05. However, among the entropy values, higher values being indicative of higher classification accuracy, the value of model 4 was smaller than that of model 3, which indicated that model 4 was no longer better than model 3. Considering all the above, we ultimately chose the three-profile solution as the best fit for medical staff.

**Table 2:** Fit indices for latent profile analysis of medical staff psychological symptoms.

Model	AIC	BIC	aBIC	Entropy	LMR (*p*)	BLRT (*p*)
1-Profile	29727.572	29784.040	29752.269			
2-Profile	25688.950	25779.299	25728.465	0.908	<0.001	<0.001
**3-Profile**	**23922.123**	**24046.353**	**23976.457**	**0.952**	**<0.001**	**<0.001**
4-Profile	23153.780	23311.891	23222.932	0.861	<0.001	<0.001

AIC, Akaike Information Criterion; BIC, Bayesian Information Criterion; aBIC, sample-size adjusted BIC; LMR-LRT, Lo–Mendell–Rubin adjusted LRT test; BLRT, bootstrap likelihood ratio test. Bold values indicate the AIC, BIC, and aBIC gradually decreased, indicating that the model’s fitting degree improved; the entropy calculations close to 1.0 indicate a better classification.

**Figure 1:**
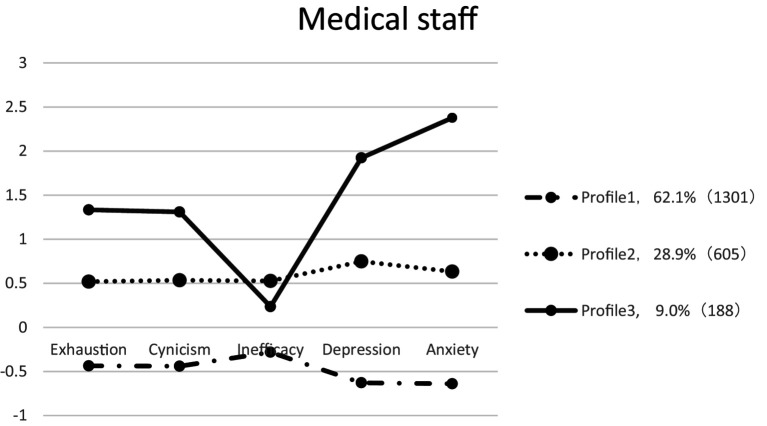
The three profiles model with Z score.

The scores on each dimension of the medical staff psychological symptom for various profiles are shown in Figure 1 and Table 3. The first and largest number of medical staff was in “Low-level” (62.1%), with lower scores for all variables, indicating that medical staff in this profile had relatively good mental health, characterized by being able to effectively solve problems at work and having a better sense of competence and accomplishment in their work. In the second profile (28.9%), medical staff members scored significantly higher than those in the first profile and were characterized by frequent feelings of sadness and high levels of negative emotions. Therefore, this profile was labeled as “Moderate-level.” The least numerous profile (9.0%) consisted of medical staff members who scored significantly much higher than the other two profiles on the dimensions of exhaustion, cynicism, depression, and anxiety. It was characterized by high levels of fatigue, difficulty at work, ease of annoyance, and irritation. Therefore, this profile was labeled as “High-level.” Notably, the dimension of the inefficacy of this profile was significantly lower than the second profile.

**Table 3:** Psychological symptoms of medical staff in different profiles.

	a. Profile 1	b. Profile 2	c. Profile 3	*F*
Medical staff				
Exhaustion	−0.434 (0.838)_b***c***_	0.524 (0.733)_a***c***_	1.319 (0.790)_a***b***_	585.217^***^
Cynicism	−0.437 (0.729)_b***c***_	0.533 (0.869)_a***c***_	1.307 (1.045)_a***b***_	468.420^***^
Inefficacy	−0.284 (1.010)_b***c***_	0.534 (0.726)_a***c***_	0.246 (0.927)_a***b***_	203.682^***^
Depression	−0.628 (0.477)_b***c***_	0.755 (0.502)_a***c***_	1.916 (0.721)_a***b***_	2404.675^***^
Anxiety	−0.640 (0.244)_b***c***_	0.637 (0.398)_a***c***_	2.376 (0.661)_a***b***_	4338.808^***^

Subscripts a–c indicate significant differences from other profiles. The psychological symptom scores with Z score. ^*^p < 0.05; ^**^p < 0.01; ^***^p < 0.001.

### Univariate analysis

Table 1 also shows the relationships between sample characteristics variables and psychological symptom profiles. Work unit, gender, marital status, and parenting status were found to be significant among the three profiles; age, job title, income, salary satisfaction, work hours, and work intensity were also statistically significant with profile membership.

### Multinomial logistic regression

The “Low-level” profile was the most prevalent group in the present study. Therefore, we used it as the reference class. At the same time, significant variables from the univariate analyses are included in the multinomial logistic regression analyses.

The results of the multinomial logistic regression analysis of variables associated with the psychological symptom profiles are listed in Table 4. There were significant differences among the latent profile members in terms of gender, age, salary satisfaction, work hours, and work intensity. Gender [OR = 1.641, 95% confidence interval, CI (0.220, 0.771)] was a predictor of “Moderate-level” profile membership. Males had a greater rate of belonging to “Moderate-level” profile members than females; age [OR = 0.881, CI (−0.224, −0.030)] also was a predictor of membership in the “Moderate-level” profile. Relatively young individuals were more likely to be members of the “Moderate-level” profiles. Work hours [OR = 1.511, CI (0.183, 0.643)] were a predictor of membership in the “High-level” profile. Low salary satisfaction [OR = 0.569, CI (−0.673, −0.456); OR = 0.644, CI (−0.616, −0.263)] and intensive work individuals [OR = 1.519, CI (0.299, 0.536); OR = 1.804, CI (0.377, 0.803)] were more likely to be members of the “Moderate-level” and “High-level” profiles.

**Table 4:** Coefficients from the multinomial logistic regression model.

Variable	Comparison of latent profiles							
	2 vs. 1				3 vs. 1			
	*B*	SE	*p*	OR	*B*	SE	*p*	OR
Work unit	0.018	0.060	0.764	1.018	0.009	0.097	0.928	1.009
Gender	0.495	0.141	<0.001	1.641	−0.149	0.192	0.437	0.861
Marital status	0.051	0.169	0.761	1.053	−0.129	0.264	0.625	0.879
Parenting status	−0.178	0.092	0.054	0.837	−0.142	0.145	0.326	0.867
Age	−0.127	0.050	0.010	0.881	−0.091	0.072	0.209	0.913
Job title	−0.003	0.095	0.973	0.997	−0.042	0.142	0.766	0.959
Income	0.023	0.057	0.685	1.023	−0.128	0.113	0.257	0.880
Salary satisfaction	−0.564	0.055	<0.001	0.569	−0.439	0.090	<0.001	0.644
Work hours	0.063	0.088	0.474	1.065	0.413	0.117	<0.001	1.511
Work intensity	0.418	0.060	<0.001	1.519	0.590	0.109	<0.001	1.804

The reference category for the dependent variables was the “Profile1, Low-level”; OR Odds ratio.

## Discussion

The present research identified the profiles and characteristics of the psychological symptoms of Chinese medical staff with frontline anti-epidemic experience through LPA based on burnout (exhaustion, cynicism, and inefficacy), depression, and anxiety symptoms. Then, the demographic and work characteristics with different psychological symptom profiles were explored.

Three latent profiles were identified, which were “Low-level,” “Moderate-level” and “High-level.” More than half of medical staff (62.1%) fell within the “Low-level” profile, with the lowest scores on all symptoms and dimensions than other profiles. The percentage of members in the “Moderate-level” profile was 28.9% in our total sample, with each score of the symptoms and the dimensions significantly higher than those of the “Low-level” profile. The “High-level” profile accounted for the lowest proportion of medical staff (9.0%), with most of the scores of the symptoms and the dimensions significantly higher than those of the “Moderate-level” and “High-level” profiles. It seems that, after the pandemic, majority of frontline medical staff in China did not face a high risk of psychological problems. However, the cultural stigma surrounding mental health issues in China may significantly affect individuals’ willingness to disclose mental health problems. The traditional emphasis on family reputation, social harmony, and personal resilience often fosters reluctance to seek help or openly discuss mental health concerns (59).

Post-hoc comparisons revealed the significant differences observed between profiles. It supported the conclusion that inherent heterogeneity exists in the psychological symptoms of medical staff. However, compared to other symptoms, members in the “High-level” profile scored very low in the inefficacy dimension of burnout, which was even lower than those in the “Moderate-level” profile. Firstly, this result was consistent with some other LPA studies focused on burnout symptoms of healthcare staff before and during the pandemic (33, 60). These results may imply that, for the worst burnout members, inefficacy was always not the typical symptom, regardless of the stress situations. However, a meta-analysis based on studies of Chinese doctors’ burnout demonstrated that inefficacy (termed as reduced personal accomplishment) was the most prevalent symptom (66.53%) among Chinese doctors before and during the pandemic (46). Given a certain amount of intensity and duration of stress situations, one symptom may increase or transfer to other psychological symptoms, causing one to experience significant emotional distress and physical illness (33, 61). This may remind us not to underestimate the potential threat of inefficacy experience to medical staff’s psychological health even if it is only at a moderate level. On the other hand, anxiety was the most prominent symptom within the “High-level” profile. As previous evidence has already shown that anxiety was the most prevalent symptom among medical staff during the COVID-19 pandemic (62), we may conclude that anxiety was not only the most prominent but also the most persistent symptom for the vulnerable medical staff, and therefore deserves the maximum concerns and interventions during and after a long-term high-pressure work.

Concerning the relationship between burnout, depression, and anxiety, our results suggest that burnout may share some common characteristics with depression and anxiety at low and moderate levels. In particular, the experiences of exhaustion and cynicism in burnout have consistently shown a similar developmental trajectory to depressive and anxiety symptoms. However, they do not develop in tandem when the symptoms worsen. This is especially true of the conclusion on the inefficacy dimension of burnout. Previous research addressing the burnout-depression or burnout-anxiety relationship, using a variable-centered approach, has already reached a consensus of a remarkable association between burnout and the other two symptoms but debating on the distinct construct of burnout (12, 14, 24). Adopted a person-centered approach, our findings expand knowledge on burnout and pervasive negative affectivity as underlying various symptom profiles. On one hand, our results suggest that burnout, anxiety, and depression are closely related; on the other, each has its unique characteristics. This provides a plausible explanation for why the debate as to whether or not burnout overlaps with depression or anxiety continues to this day (13, 14, 63, 64). In sum, frontline medical staff may experience various patterns of psychological symptoms, with the relationship of the three burnout dimensions and its association with depression, and anxiety changing at different points in time. Therefore, our findings further confirm that burnout is a distinct construct. In particular, the relationship between exhaustion and cynicism in burnout and depression and anxiety remains stronger at all stages, whereas inefficacy in burnout does not continue to worsen with the escalation of other symptoms. On one hand, the dimensions of exhaustion and cynicism have been consistently shown in various studies to be more strongly related (33). Another possible explanation is that during and after the pandemic, frontline medical staff in China were praised and celebrated as models of collectivism and patriotism, which may have helped preserve their sense of efficacy. However, the emotional exhaustion resulting from excessive workload and emotional labor, along with alienation from their work environment and patients, was unlikely to be alleviated by such social recognition.

Also, our results indicate the impact of gender, age, salary satisfaction, work hours, and work intensity on the development of psychological symptoms. Specifically, “Moderate-level” profile members were more likely to be younger than “Low-level” profile members. This could be attributed to the workload and lack of working experience at the beginning of young medical staff careers (63, 65). On the other hand, more occupational experience of older medical staff may help in developing effective strategies to cope with diverse stressors and thus maintain their resilience (66). Next, it found that male medical staff members were more likely to be “Moderate-level” profile members than “Low-level” profile members, indicating that male medical staff members were more likely to experience severe psychological symptoms than females after the pandemic, which was inconsistent with both the results of previous person-centered approaches and variable-centered approaches (67–69). This may be related to gender differences in coping styles with negative emotions (70). At the same time, this discrepancy could be related to various assessment scales used, different samples selected, and different data analyses used in these studies. In addition, due to convenient sampling in this study, a relatively small sample size of male frontline medical staff might lead to cases of bias, so the conclusions need to be further validated.

In addition, frontline medical staff members who faced lower salary satisfaction, longer work hours, and greater work intensity were more likely to be “Moderate-level” or “High-level” profile members. These results were aligned with previous psychological health results based on the variable-centered approach, which showed a relationship between salary satisfaction, work hours, work intensity, and psychological health level (9, 71, 72). These findings suggest that hospital administrators can effectively identify medical staff with poor psychological health through demographic and work-related characteristics, and implement appropriate interventions to reduce their psychological symptoms.

Furthermore, this research has several strengths. Firstly, a large sample size was collected with a sufficient response, which provided adequate data support for the study. Secondly, the latent profile analysis method was used to investigate medical staff characteristics in the post-COVID-19 epidemic era, which effectively differentiated the heterogeneity of medical staff’s psychological symptoms, facilitating an understanding of the unique characteristics and latent differences of the different groups’ symptoms. Thirdly, burnout, depression, and anxiety symptoms were assessed jointly to explore their relationship with a person-centered approach.

The study adopted a person-centered method to explore the group heterogeneity of psychological symptoms among medical staff, but several limitations of the current study should be noted. Firstly, this study was based on a cross-sectional design, which limits the ability to establish causal relationships. Longitudinal studies, incorporating a greater number of observational sites, are needed to examine the dynamic progression of psychological symptoms. Latent transition analysis could be employed to explore changes in latent profiles over time, while cross-lagged analysis may be used to investigate the interactions or reciprocal relationships between these symptoms. Secondly, this study was conducted solely in China, which may limit the generalizability of the findings to other cultural contexts. Thirdly, data were collected through self-report questionnaires, which are susceptible to social desirability bias and common method variance. To mitigate these limitations, future research should incorporate both objective and subjective indicators in measuring psychological symptoms. Additionally, inter-rater reliability statistics could be utilized to enhance the reliability of the data. Fourthly, the sampling in our study was voluntary and conducted through an online platform, which introduces the potential for selection bias. Finally, this study focused solely on the effects of demographic and work-related variables. Future studies should investigate potential moderators or protective factors (e.g., coping strategies, social support) to deepen the understanding of the relationship between work-related factors and mental health, thereby facilitating the design and implementation of future intervention programs.

## Conclusion

In summary, our findings revealed that, in the post-COVID-19 pandemic era, burnout (exhaustion, cynicism, and inefficacy), depression, and anxiety symptoms of Chinese medical staff with frontline anti-epidemic experience could be categorized into three profiles, which were defined as “Low-level,” “Moderate-level,” and “High-level.” Symptoms of burnout, depression, and anxiety did not move in lock-step, which supports the proposition that burnout is a distinct construct. In the low-level and moderate-level profiles, all the symptoms and dimensions considered had a consistent trend. However, within the high-level profiles, the inefficacy dimension of burnout remained at a lower level, and anxiety performed as the most prominent symptom. For medical staff, gender, age, salary satisfaction, work hours, and work intensity significantly influence the latent profiles of psychological symptoms among medical staff.

Therefore, the work-related pressures of frontline medical staff with anti-epidemic experience deserve more concern even in the post-COVID-19 pandemic era, for the prevention of not only burnout but also depression and anxiety. Meanwhile, more care and intervention measures should be targeted at men and young ones. For those medical staff with the worst mental health conditions, interventions should focus on symptoms of anxiety and depression directly. On the contrary, interventions targeting the inefficacy of burnout may not be effective. Future longitudinal and cross-cultural studies are needed to validate the findings of this research and provide insights for interventions.

## Data Availability

The raw data supporting the conclusions of this article will be made available by the authors, without undue reservation.
